# Unconventional activation of PRKDC by TNF-α: deciphering its crucial role in Th1-mediated inflammation beyond DNA repair as part of the DNA-PK complex

**DOI:** 10.1186/s12950-024-00386-x

**Published:** 2024-04-30

**Authors:** Mohamed A. Ghonim, Jihang Ju, Kusma Pyakurel, Salome V. Ibba, Mai M. Abouzeid, Hamada F. Rady, Shigemi Matsuyama, Luis Del Valle, A. Hamid Boulares

**Affiliations:** 1 The Stanley S. Scott Cancer Center, LSU Health Sciences Center-New Orleans, 1700 Tulane Ave, New Orleans, LA 70112 USA; 2 Department of Microbiology and Immunology, Faculty of Pharmacy, Al-Azhar University, Cairo, Egypt; 3 Department of Ophthalmology and Visual Science; Case Comprehensive Cancer Center, Case Western Reserve University, Cleveland, OH USA

**Keywords:** Inflammation, PRKDC, DNA repair, TNF-α, Phosphorylation

## Abstract

**Background:**

The DNA-dependent protein kinase (DNA-PK) complex comprises a catalytic (PRKDC) and two requisite DNA-binding (Ku70/Ku80) subunits. The role of the complex in repairing double-stranded DNA breaks (DSBs) is established, but its role in inflammation, as a complex or individual subunits, remains elusive. While only ~ 1% of PRKDC is necessary for DNA repair, we reported that partial inhibition blocks asthma in mice without causing SCID.

**Methods:**

We investigated the central role of PRKDC in inflammation and its potential association with DNA repair. We also elucidated the relationship between inflammatory cytokines (e.g., TNF-α) and PRKDC by analyzing its connections to inflammatory kinases. Human cell lines, primary human endothelial cells, and mouse fibroblasts were used to conduct the in vitro studies. For animal studies, LPS- and oxazolone-induced mouse models of acute lung injury (ALI) and delayed-type hypersensitivity (DHT) were used. Wild-type, PRKDC^+/−^, or Ku70^+/−^ mice used in this study.

**Results:**

A ~ 50% reduction in PRKDC markedly blocked TNF-α-induced expression of inflammatory factors (e.g., ICAM-1/VCAM-1). PRKDC regulates Th1-mediated inflammation, such as DHT and ALI, and its role is highly sensitive to inhibition achieved by gene heterozygosity or pharmacologically. In endothelial or epithelial cells, TNF-α promoted rapid PRKDC phosphorylation in a fashion resembling that induced by, but independent of, DSBs. Ku70 heterozygosity exerted little to no effect on ALI in mice, and whatever effect it had was associated with a specific increase in MCP-1 in the lungs and systemically. While Ku70 knockout blocked VP-16-induced PRKDC phosphorylation, it did not prevent TNF-α − induced phosphorylation of the kinase, suggesting Ku70 dispensability. Immunoprecipitation studies revealed that PRKDC transiently interacts with p38MAPK. Inhibition of p38MAPK blocked TNF-α-induced PRKDC phosphorylation. Direct phosphorylation of PRKDC by p38MAPK was demonstrated using a cell-free system.

**Conclusions:**

This study presents compelling evidence that PRKDC functions independently of the DNA-PK complex, emphasizing its central role in Th1-mediated inflammation. The distinct functionality of PRKDC as an individual enzyme, its remarkable sensitivity to inhibition, and its phosphorylation by p38MAPK offer promising therapeutic opportunities to mitigate inflammation while sparing DNA repair processes. These findings expand our understanding of PRKDC biology and open new avenues for targeted anti-inflammatory interventions.

**Supplementary Information:**

The online version contains supplementary material available at 10.1186/s12950-024-00386-x.

## Background

DNA-PK is critical for repairing double-stranded DNA breaks (DSBs) through nonhomologous end-joining (NHEJ) [1, 22]. It is composed of a 450-kDa catalytic subunit (PRKDC) and two DNA-binding subunits (Ku70/Ku80). Ku proteins are required for the function of the kinase in DNA repair. Once activated, PRKDC phosphorylates several proteins, including itself, p53, histones (e.g., H2AX), and Ku70/Ku80 [1]. These events organize the trafficking of other enzymes (e.g., Artemis endonuclease, XRCC4, DNA ligase, etc.) to DSBs [1, 22]. Deficiency in PRKDC (or the Ku70/80 proteins) causes SCID [24]. Since its discovery almost three decades ago, it has not been clear why humans and many other mammals express very high levels of PRKDC when less than 1% is needed to repair DNA damage [22, 45]. The high levels of PRKDC in human cells are paradoxical because this does not confer any additional capacity in repairing DNA damage [22]. If DNA-PK does not endow human cells with particular resistance to DNA damage, why do human cells universally express such high levels of its catalytic subunit PRKDC?

We reported that PRKDC is required for TNF-α or IL-1β-induced NF-κB-dependent gene expression (e.g., VCAM-1), in part, by phosphorylating p50 NF-κB on a single serine residue (Ser20) [17]. We demonstrated that *PRKDC* heterozygosity, which reduces the protein level and kinase activity by ~ 50%, blocks asthma [8] through the regulation of *gata-3* expression without causing SCID. We initially speculated that the role of PRKDC in inflammation is strictly associated with oxidative stress-generated DSBs. However, because of the strong connection between SCID and the inability of immune cells to perform NHEJ, the results [8] suggested that the role of PRKDC in Th2-associated inflammation was independent of DSB repair. We thus reassessed our hypothesis to surmise that the kinase complex, primarily PRKDC, functions independently of its classical role in DNA repair. It is noteworthy that Ku70 and Ku80 have been reported to exhibit noncanonical roles in inflammation, including asthma, but are still related to DNA damage responses [34].

The potential DNA repair-independent feature of PRKDC may amount to a new function for the kinase, perhaps as a novel enzyme. This may constitute a yet to be explored therapeutic target against inflammatory processes and diseases. It is important to note that the major issue of targeting single enzymes to modulate inflammation is the fact that often inhibiting the targets would also negatively affect their physiological roles, hence the potential emergence of side effects [38]. As stated above, the role of the DNA-PK complex in DNA repair, NHEJ, and immunoglobulin generation requires a minute level of PRKDC [22]. Reducing PRKDC by ~ 50% does not affect the role of the kinase in T and B-cell generation, as PRKDC^+/−^ mice behave as wild-type mice with intact T and B-cell populations [8]. On the other hand, the role of the kinase in inflammation is extremely sensitive to modulation, as a mere 50% reduction in PRKDC by gene heterozygosity protects against asthma in mouse models of the disease [8].

Acute lung injury (ALI) is characterized by severe, Th1-mediated inflammatory responses within the lung, resulting in diffuse damage to the alveolar-capillary barrier and causing an influx of many inflammatory cells, namely, neutrophils, macrophages, and lymphocytes [35, 42]. A prominent model for ALI is that promoted by exposure in the lung to bacterial lipopolysaccharide (LPS). Delayed-type hypersensitivity (DHT) models such as oxazolone-induced contact DHT are also excellent models of Th1-mediated inflammatory diseases. The following study was designed to address the underlying mechanism(s) by which a DNA repair enzyme (PRKDC) participates in molecular events in response to inflammatory cues, primarily TNF-α, and separate this role in inflammation from its canonical role in DNA repair with the aim of establishing the catalytic subunit as a unique enzyme whose principle function is in inflammation.

## Methods

### Animals, treatments, organ recovery, human samples, and analysis of tissues and fluids

C57BL/6 J wild type (WT) mice were purchased from Jackson Laboratory (Bar Harbor, ME). PRKDC^+/−^ mice on a C57BL/6 J genetic background [8] and Ku70^+/−^ mice backcrossed onto a C57BL/6 J background for at least 5 generations were bred and maintained in a specific-pathogen free facility at Louisiana State University Health Sciences Center of New Orleans. Mice had unlimited access to sterilized chow and water. All experimental protocols and procedures were approved by the IACUC. For oxazolone-induced ear DTH, ~ 6-week-old WT or PRKDC^+/−^ mice were sensitized through topical application of 150 μl of 3% oxazolone (Sigma‒Aldrich) in acetone onto their shaved abdomens on day 1. On day 2, ear thickness was measured and considered baseline. Ear thickness measurements were taken with a digital caliper (Mitutoyo). On day 6, mice were challenged through application of 20 μl of 3% oxazolone in acetone or vehicle to the inner and outer surfaces of both ears. Some WT mice were administered 5 mg/kg NU-7441 (Tocris Bioscience) *i.p.* 30 min after challenge. Edema was calculated for each ear as the difference in thickness before treatment (0 h) and 48 h after challenge. For the ALI model, ~ 6-week-old mice received intratracheal instillation of saline or LPS (Sigma‒Aldrich) at a dose of 10 mg/kg. Some WT mice received an intranasal administration of NU7441 at a dose of 5 mg/kg or vehicle 30 min after exposure to LPS followed by a second administration, intraperitoneally, 8 h later. Mice were euthanized 18 h after LPS treatment for serum and tissue collection or bronchoalveolar lavage fluid (BALF) for cell counting and cytokine assessment. Inflammatory cells in BAL fluids were stained with diff-quick staining. Cytokine assessment was performed using Milliplex MAP kits (MilliporeSigma) for mouse cytokines. The lung wet-to-dry (W/D) weight ratio, an index of lung edema, was conducted as described [20]. Pulmonary neutrophil sequestration was quantified using a myeloperoxidase assay (MPO) assay as outlined by the manufacturer’s instructions (Enzo-Life Sciences). Two de-identified lung specimens from individuals who died of severe asthma, two from individuals who died from acute respiratory distress syndrome (ARDS) and two individuals who died from lung disease-unrelated causes were acquired from the LSUHSC Pathology Department. Sections from these samples were subjected to IHC, as previously described [27], with antibodies to the phosphorylated form of PRKDC at S2056 (Abcam, Waltham, MA).

### Cell culture, treatments, RT‒PCR, knockdown with siRNA, immunoprecipitation (IP), immunoblot analysis, and cell-free phosphorylation reactions

WT, PRKDC^+/−^, and PRKDC^−/−^ HCT-116 cells were purchased from Horizon Discovery. U937 cells were purchased from ATCC. Human aortic endothelial cells were purchased from Lonza Bioscience. Mouse embryonic fibroblasts (MEFs) were isolated from embryos of WT or Ku70^−/−^ mice [30]. Cells were treated as indicated in the figure legends with TNF-α (R&D Systems), VP-16 (Sigma‒Aldrich), H_2_O_2_ (Sigma‒Aldrich), or LPS (Sigma‒Aldrich). Some cells were treated with a variety of drugs, most of which were purchased from Selleckchem. For immunoblot analysis, whole cell extracts were prepared using standard protocols. The antibodies used in this study were against the following: PRKDC, phosphorylated form of PRKDC at S2056 or S2612, or ICAM-1 were from Abcam; p53, phosphorylated form of p53 at S15 or S37, γH2AX, p38MAPK or its phosphorylated form at T80/Y182, p65 NF-κB or its phosphorylated form at S536; I-κBα or its phosphorylated form at S32; Ku70, VCAM-1, β-Actin and GAPDH were from Santa Cruz Biotechnology. Immunoprecipitation was conducted essentially as described [17]. Real-time and conventional PCRs were conducted using standard protocols with primers specific to mouse or mouse *vcam-1* or human *icam-1* as described [8, 17]. Knockdown of Ku70 in HAECs was achieved using siRNA(h) (sc-29383; Santa Cruz Biotechnology) or scrambled sequence (control) according to the manufacturer’s instructions.

### Cell-free kinase assay

Purified DNA-PK complexes (PRKDC/Ku70/Ku80) isolated from HeLa cells (Promega) and recombinant human active p38MAPKα (R&D Systems) were incubated in a kinase reaction as described [17]. In some experiments, [γ-^33^P]ATP (PerkinElmer Life Sciences) was used instead of ATP. The samples were resolved using SDS‒PAGE followed by autoradiography or subjected to immunoblot analysis with the indicated antibodies.

### Data analysis

GPower 3.1.9.2 software was used to determine the sample size required to estimate mean differences between two independent groups. A sample size of 6 mice/genotype/treatment group/time point was expected to yield 95% power to detect statistically significant differences with *p* values of 0.01–0.05. All data are expressed as the mean ± SEM of values from triplicates samples for qPCR or mouse experimental groups (*n* ≥ 6). The in vitro experiments were conducted at least three times. PRISM software (GraphPad) was used to analyze the differences between experimental groups by one-way ANOVA followed by Tukey method.

## Results

### Partial reduction of PRKDC is sufficient to block the TNF-α-mediated inflammatory response in human cells and protects against oxazolone-induced contact DTH or LPS-mediated acute lung injury in mice

**Fig. 1:**
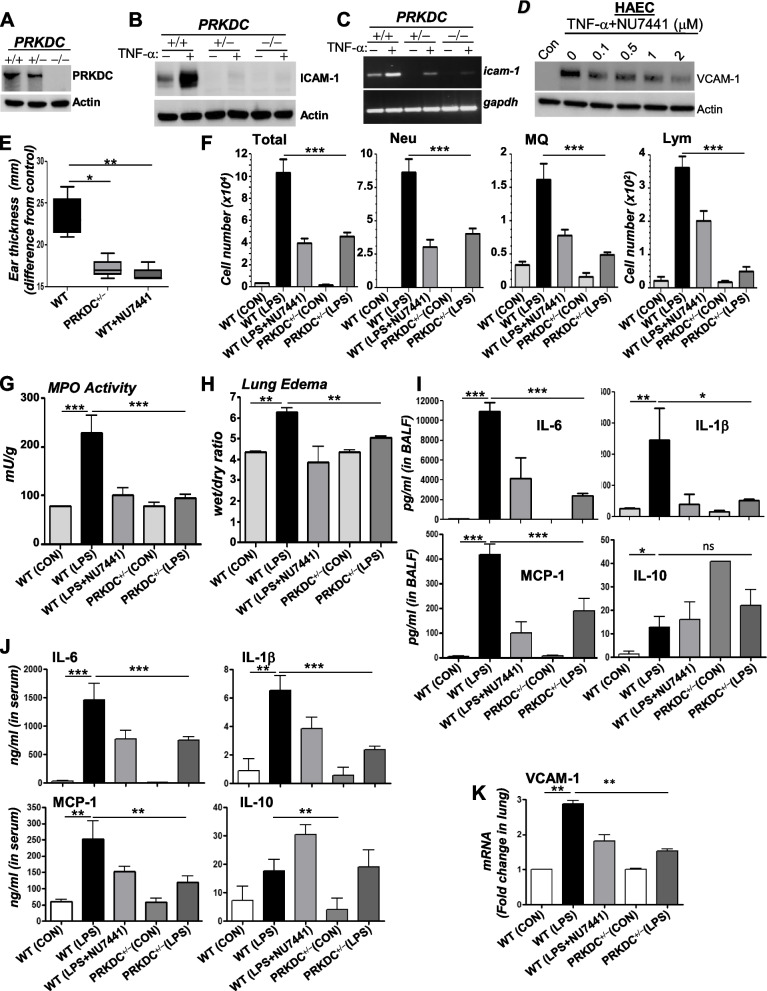
Effects of partial reduction of PRKDC on TNF-α-induced ICAM-1 expression in human cells and oxazolone-induced contact DTH or LPS-mediated ALI in mice. **A** Immunoblot analysis of protein extracts from HCT116 cells of different genotypes with antibodies against PRKDC or actin. **B** Cells were treated with TNF-α (10 ng/ml) for 24 h; protein extracts were assessed for ICAM-1. **C** Cells were treated as in (**B**) but for 6 h. RT‒PCR with primers specific to human *icam-1* or *gapdh*. **D** Endothelial cells were treated with TNF-α with or without NU7441. Eighteen hours later, protein extracts were assessed for VCAM-1 or actin. **E** WT and PRKDC^+/−^ mice were sensitized to oxazolone on day 1. Challenge was performed on day 6 through application of 20 μl of 3% oxazolone in acetone to both ears. NU7441 (5 mg/kg) was administered after challenge. Ear thickness measurements were then measured. Edema was calculated for each ear as the difference in thickness before treatment (0 h) and 48 h after challenge. **F** Mice were administered, *i.t.,* 10 μg/kg LPS or saline. Some WT mice received NU7441 i.p. after LPS exposure. Mice were sacrificed 18 h later. Cells of BAL fluids were differentially stained, and total cells, neutrophils (Neu), macrophages (MQ), and lymphocytes (Lym) were counted. **G** Lung homogenates were measured for myeloperoxidase (MPO). **H** Pulmonary edema was measured using the weight/dry ratio. Cytokine levels were measured in BALF (**I**) or sera of animals (**J**) using Multiplex kits. Data are expressed as the means ± SDs of values from *n* ≥ 6 per group. *, **, ***, difference from LPS-treated WT animals with *p* ≤ 0.05, *p* ≤ 0.01. *p* ≤ 0.01, respectively. **K** Total RNA from whole lungs was subjected to reverse transcription followed by real-time PCR with primers specific to mouse *vcam-1*. Data are means ± SDs of values from triplicates. *, **, ***, difference from TNF-α-treated cells with *p* ≤ 0.05, *p* ≤ 0.01. *p* ≤ 0.01, respectively

We have shown that partial knockdown of PRKDC in human endothelial cells and PRKDC heterozygosity in mouse lung smooth muscle cells markedly reduces TNF-α-induced expression of the adhesion molecule VCAM-1, suggesting that the manifestation of inflammation is very sensitive to changes in PRKDC levels [8]. Given that human cells exhibit higher PRKDC activity than other mammals [6, 22], we wished to precisely determine whether a ~ 50% reduction in PRKDC in human cells affects the manifestation of inflammation. To this end, the human cancer cell line HCT116 with isogenic heterozygosity or knockout of PRKDC ([36] and Fig. 1A) was assessed for its response to TNF-α to express the adhesion molecule ICAM-1 as a readout of inflammation. Treatment with TNF-α induced a substantial amount of ICAM-1, and PRKDC heterozygosity was sufficient to almost completely block the expression of the adhesion molecule at the protein (Fig. 1B) and mRNA (Fig. 1C) levels in response to this treatment. Such blockade is not temporary, as extended treatment with TNF-α did not result in an increase in ICAM-1 expression in PRKDC^+/−^ HCT116 cells (Supplementary Fig. S1A). TNF-α-induced expression of inflammatory factors (e.g., VCAM-1) in human endothelial cells (HAECs) was equally highly sensitive to pharmacological inhibition of DNA-PK by NU7441 (Fig. 1D). These results establish that the manifestation of inflammation may be highly sensitive to PRKDC levels.

We previously showed that PRKDC heterozygosity or moderate treatment with the inhibitor NU7441 markedly protected against asthma-like traits in two animal models of the disease, in part, through the inhibition of Th2 cytokines [8]; however, some Th1 cytokines were affected. This led us to speculate that DNA-PK may be a more general regulator of inflammatory responses and diseases. We therefore examined the role of the kinase in two inflammatory diseases, DTH and ALI, which are primarily driven by Th1 responses. DTH is a type IV cell-mediated hypersensitivity primarily by Th1 cells [43] and represents a good model for Th1 diseases. Unlike asthma, ALI is primarily mediated by the recruitment of neutrophils to the lung in response to infections or bacterial byproducts such as LPS.

Figure 1E shows that WT mice that were subjected to oxazolone treatments developed a marked swelling of the ears, a measure of inflammatory edema, and that PRKDC heterozygosity or a single treatment with NU7441 almost completely blocked such swelling after drug challenge. As shown in our previous work [46], intratracheal administration of LPS in WT mice induced massive neutrophilia of the lung, which was significantly reduced in the lungs of similarly exposed PRKDC^+/−^ mice (Fig. 1F). The increase in macrophages and lymphocytes in LPS-treated WT mice was also reduced in their PRKDC^+/−^ counterparts (Fig. 1F). An assessment of myeloperoxidase (MPO), another measure of neutrophilia, also demonstrated the potent anti-inflammatory effects of PRKDC heterozygosity and treatment with NU7441 (Fig. 1G). Lung edema, which is characterized by increased permeability of the alveolar-capillary barrier, represents an important aspect of ALI [16]. PRKDC heterozygosity or treatment with NU7441 prevented LPS-induced lung edema in mice (Fig. 1H). The anti-inflammatory effects of PRKDC inhibition in LPS-treated mice were accompanied by substantial decreases in the levels of the proinflammatory cytokines IL-6, IL-1β, and MCP-1 in BALF, with a slight increase in the anti-inflammatory cytokine IL-10 (Fig. 1I). The levels of the aforementioned cytokines/chemokines followed relatively similar patterns in sera of the different experimental groups (Fig. 1J). An assessment of mRNA levels in the lungs of the different experimental groups showed that DNA-PK modulation affected *IL-6*, *MCP-1*, and *IL-1β* expression (Supplemental Fig. S2). The effects of DNA-PK inhibition on inflammatory cells may also be related to a concomitant reduction in *VCAM-1* expression (Fig. 1K).

Altogether, the above results as well as those previously reported by us [8, 17] and others [25] provide compelling evidence that PRKDC plays a critical role in different forms of inflammation. More importantly, the role of the kinase in inflammation requires high levels of its catalytic subunit, and a reduction as low as 50% dramatically blocks the manifestation of these conditions. Conversely, however, the role of PRKDC in DNA repair requires very low levels of the kinase [22, 24]. Based on this interesting conundrum, an important question is whether PRKDC is activated during inflammation and whether such activation involves DNA damage.

### PRKDC modification at either the ABCDE (S2612) or the PQR (S2056) phosphorylation clusters is highly sensitive to TNF-α treatment and is prevalent in sites of inflammation in human asthma and acute lung injury (pneumonia)

**Fig. 2:**
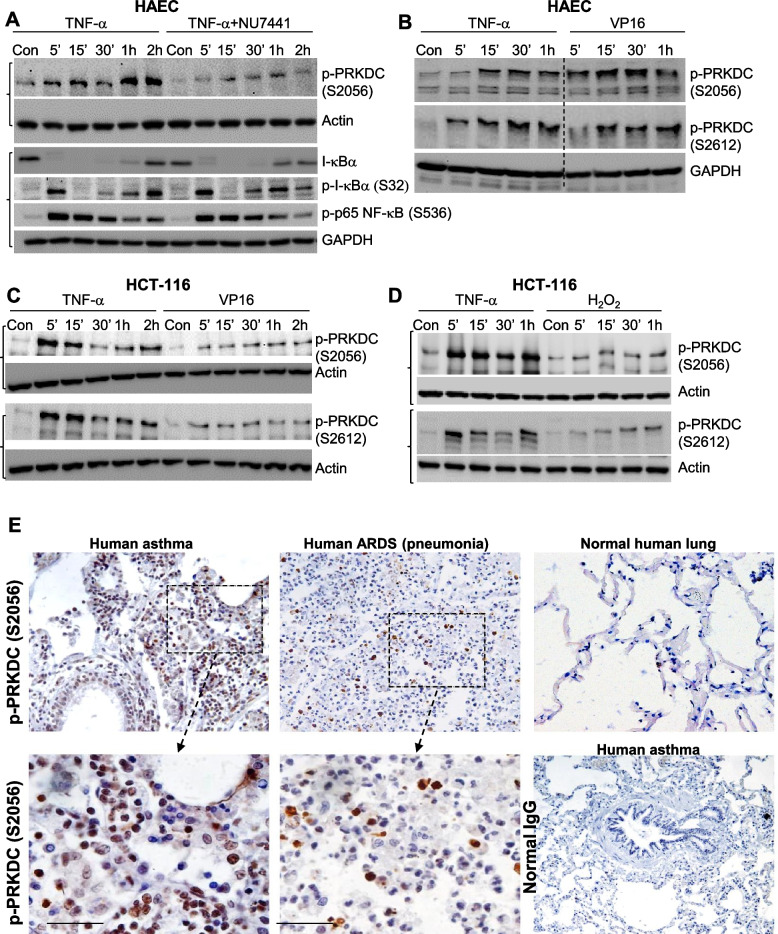
TNF-α-mediated induction of PRKDC phosphorylation at the ABCDE (S2612) and PQR (S2056) clusters and its prevalence in the lungs of asthmatic patients and individuals with acute lung injury. **A** HAECs were treated with 10 ng/ml TNF-α in the presence or absence of 2 μM of the DNA-PK inhibitor NU7441 for the indicated time intervals. Protein extracts were then subjected to immunoblot analysis with antibodies against the phosphorylated forms of PRKDC (S2612 or S2056) or actin. The same samples were also analyzed for I-κBα, phosphorylated I-κBα, p65 NF-κB, and phosphorylated p65 NF-κB. **B** HAECs were treated with TNF-α or 20 μM VP-16 for the indicated time intervals. Protein extracts were subjected to immunoblot analysis with antibodies against phosphorylated PRKDC or GAPDH. HCT116 cells were treated with TNF-α or VP-16 (**C**) or TNF−α or H_2_O_2_ (**D**), and protein extracts were subjected to immunoblot analysis. Brackets indicate the same samples but run on a different gel. **E** Tissue sections from individuals who died from asthma, ARDS, or a condition unrelated to lung disease (normal) were subjected to immunohistochemistry with antibodies against p-S2056 and PRKDC. A section from an asthmatic patient was stained with normal IgG as a control

In the DNA repair process, the Ku subunits recognize DSBs and recruit upon phosphorylation to form multimeric DNA-PK complexes on both ends of the DSBs [24]. While phosphorylation of PRKDC at S2612, a site within a conserved phosphorylation ABCDE cluster, promotes access to DNA ends, phosphorylation of the kinase at S2056, a site within an equally conserved PQR phosphorylation cluster, allows the kinase to be released from DNA ends [29], although it was reported to be dispensable for lymphocyte development and class switch recombination [15]. We examined whether inflammatory factors induce modification of either or both of these sites. We selected TNF-α as a representative cytokine for its prevalence in most inflammatory responses, including ALI [19]. Figure 2A shows that TNF-α induced a rapid (as early as 5 min) and pronounced phosphorylation pattern at both the S2056 and S2612 sites in primary human endothelial cells (HAEC) as assessed by immunoblot analysis with antibodies specific to the respective sites. TNF-α-induced PRKDC phosphorylation was markedly reduced upon the addition of its inhibitor NU7441 without affecting NF- κB signaling, suggesting that this is not a general effect on TNF-α signaling. Figure 2B shows that the pattern of TNF-α-induced PRKDC phosphorylation at the S2056 and S2612 sites was similar to that induced by VP-16 (etoposide), a topoisomerase inhibitor and a DNA damaging agent. TNF-α-induced phosphorylation of PRKDC was not limited to endothelial cells, as it was induced in the epithelial cancer cell line HCT116 (Fig. 2C-D). However, the pattern of phosphorylation at these two sites was more pronounced than that induced by either VP-16 or H_2_O_2_.

To support the findings obtained using animal models of diseases and cell culture systems, a demonstration that PRKDC is indeed activated in human diseases is needed. To this end, we examined the phosphorylation status of the kinase in human specimens collected from two individuals who died from severe asthma and two who died from pneumonia-associated ARDS and compared them to sections from two individuals who died from lung disease-unrelated causes. Tissue sections were subjected to immunohistochemistry with antibodies against the phosphorylated form of PRKDC at S2056. Figure 2E shows that PRKDC was highly phosphorylated in the diseased tissues and was prevalent in many different cell types, including endothelial and epithelial cells. Lung sections from normal individuals did not display any obvious PRKDC phosphorylation (Fig. 2E, rightmost panel). The use of normal rabbit IgE for IHC showed no immunoreactivity. These qualitative results, along with the protective effect of DNA-PK inhibition against asthma [8], ALI, and DTH, demonstrate the possibility that the kinase is a component of disease manifestation and potentially a driver of inflammatory conditions.

### TNF-α-induced PRKDC phosphorylation is not associated with either the generation of DSBs or culminates in the phosphorylation of the DNA damage-related substrates p53 and H2AX; in fact, DNA damage may interfere with the expression of inflammatory factors

**Fig. 3:**
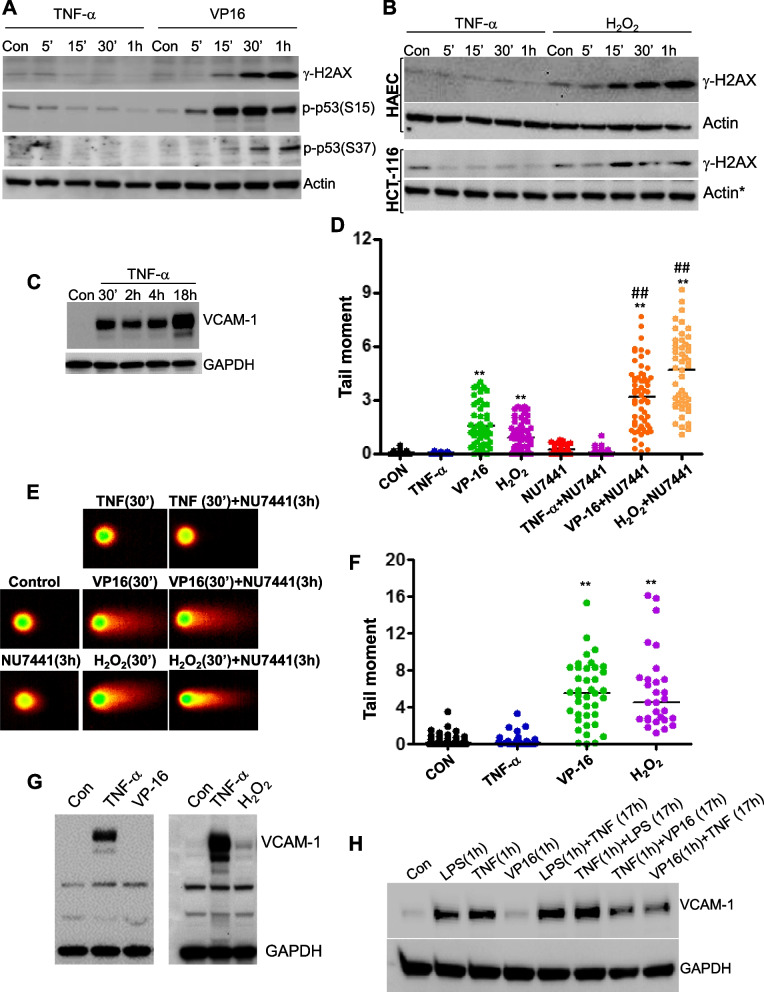
TNF-α-induced PRKDC phosphorylation is not related to DNA damage responses. **A** HAECs were treated with 10 ng/ml TNF-α or 20 μM VP-16 (**A**) or TNF-α or 100 μM H_2_O_2_ (**B**) as described in Fig. 2. γH2AX or phospho-p53 (S15 or S37) were determined by immunoblot analysis with antibodies specific to the modified proteins or actin. * indicates that the actin blot is the same as in Fig. 2D, bottom panels. **C** HAECs were treated with TNF-α for 30 min, 2 h, or 4 h, after which the medium was replaced with fresh medium without the cytokine or left unchanged. Protein extracts were prepared 18 h later for immunoblot analysis with antibodies against VCAM-1 or GAPDH. **D** U937 cells were treated with TNF-α, VP-16 or H_2_O_2_ for 30 min without recovery. Other sets of cells were treated with the same agents but left to recover in the presence of NU7441. All cells were then analyzed by the comet assay for the generation of DSBs. The results are presented as the tail moments of comets analyzed by the LAI Automated Comet Assay Analysis System. **E** Examples of comets generated by the different experimental groups described in (**D**). **F** HAECs were treated with TNF-α, VP-16 or H_2_O_2_ for 30 min without recovery, and tail moments were assessed as described in (**D**). **G** Cells were treated with TNF-α, VP-16, or H_2_O_2_ for 12 h. Extracts were subjected to immunoblot analysis for VCAM-1 or GAPDH. **H** HAECs were treated with TNF-α, VP-16, or LPS, individually or in combination, as indicated for the indicated times and order. All cells were collected 18 after the initial treatment, and protein extracts were subjected to immunoblot analysis with antibodies against VCAM-1 or GAPDH. **, ##, *p* ≤ 0.01, differences from untreated and treated groups, respectively

Two of the most prominent responses to DNA damage are phosphorylation of the tumor suppressor p53 and histone H2AX (γH2AX), which can be mediated, in part, by DNA-PK [22]. We thus examined whether TNF-α treatment induces these events. Interestingly, while VP-16 induced robust p53 and H2AX phosphorylation, TNF-α treatment failed to induce such phosphorylation (Fig. 3A). TNF-α treatment induced little γ H2AX even after 18 h of treatment (Supplemental Fig. S3). Similar differences were attained when H_2_O_2_ was used as a DNA damaging agent in endothelial and epithelial cells (Fig. 3B). These results provide additional support for the notion that the mechanisms by which TNF-α induces PRKDC phosphorylation are different from those promoted by DNA-damaging agents and suggest that the consequences of such modification may be different. More importantly, these results imply that TNF-α does not induce DNA damage during the period of time it induces its receptor-mediated signal transduction (Fig. 2A). Notably, exposure to TNF-α for 30 min was sufficient to induce the expression of substantial VCAM-1 levels in endothelial cells when assessed 24 h later (Fig. 3C).

Phosphorylation of p53 and H2AX represents a DNA damage response but not direct evidence of the existence of DSBs. We thus used the comet assay to directly demonstrate whether TNF-α treatment induces DSBs. The human monocytic cell line U937 was purposely selected because of its sensitivity to DNA-damaging agents such as VP-16 [2] and because it requires minimum handling for the assay. Cells were treated with TNF-α, VP-16 or H_2_O_2_ for 30 min. Some cells were incubated for an additional 3 h in the presence of NU7441. Cells were then processed and assessed for DSBs (*n* =  ~ 70) using the LAI Automated Comet Assay Analysis System (LACAAS). VP-16 and H_2_O_2_ treatment induced marked DNA damage, which was enhanced by NU7441 (Fig. 3D-E). However, TNF-α treatment caused no detectable DSBs. Longer treatment with TNF-α (~ 18 h) caused no significant change in tail moments (data not shown), which was confirmed by an assessment of γH2AX (supplemental Fig. S3). Treatment of cells with NU7441 did not cause any accumulation of DNA damage after 3 h of recovery. The inability of TNF-α to induce DNA damage was confirmed using human primary endothelial cells (Fig. 3F).

The above results demonstrate a lack of correlation between the DNA repair machinery and TNF-α-induced inflammation. Figure 3G provides more evidence for this conclusion, as treatment of endothelial cells with the DNA-damaging agent VP-16 or H_2_O_2_ resulted in no or little induction of VCAM-1 expression, respectively, compared to that achieved by TNF-α. We [28] and others [4] reported a potential link between DSBs and Th2-mediated inflammation. Based on this and our results, we hypothesized that if the generation of DSBs and inflammatory factors coexist, the two stimuli may cause additive or synergistic effects. To test this hypothesis, we examined the effect of VP-16 on TNF-α-induced VCAM-1 expression in endothelial cells. HAECs were treated with TNF-α for 1 h followed by treatment with VP-16, and samples were assessed 17 h later. We also conducted the reverse treatment regimen. VP-16, as expected, did not induce VCAM-1 expression (Fig. 3H); interestingly, however, it reduced TNF-α-induced VCAM-1 expression. A reduction in VCAM-1 expression was also observed when cells were subjected to the reversed treatments (i.e., 1 h of VP-16 treatment followed by ~ 17 h of TNF-α exposure). No such interference was observed when LPS, a non-DNA damaging agent that signals through TLR4, was combined with TNF-α; Instead, additive effects were observed. It is noteworthy that treatment of cells with the tested as well as higher concentrations of VP-16 for ~ 18 h with or without TNF-α did not promote substantial activation of apoptosis as assessed by the appearance of its marker, active caspase-3 (Supplemental Fig. S4); the activation of the protease was observed only when cells were treated with 20 μM VP-16. These results clearly show that the DNA damage response (i.e., DNA-PK activation) may exhibit a completely different function from that of PRKDC in inflammation.

### Unlike PRKDC, the role of Ku70 in inflammation may be dispensable or protective against inflammation

**Fig. 4:**
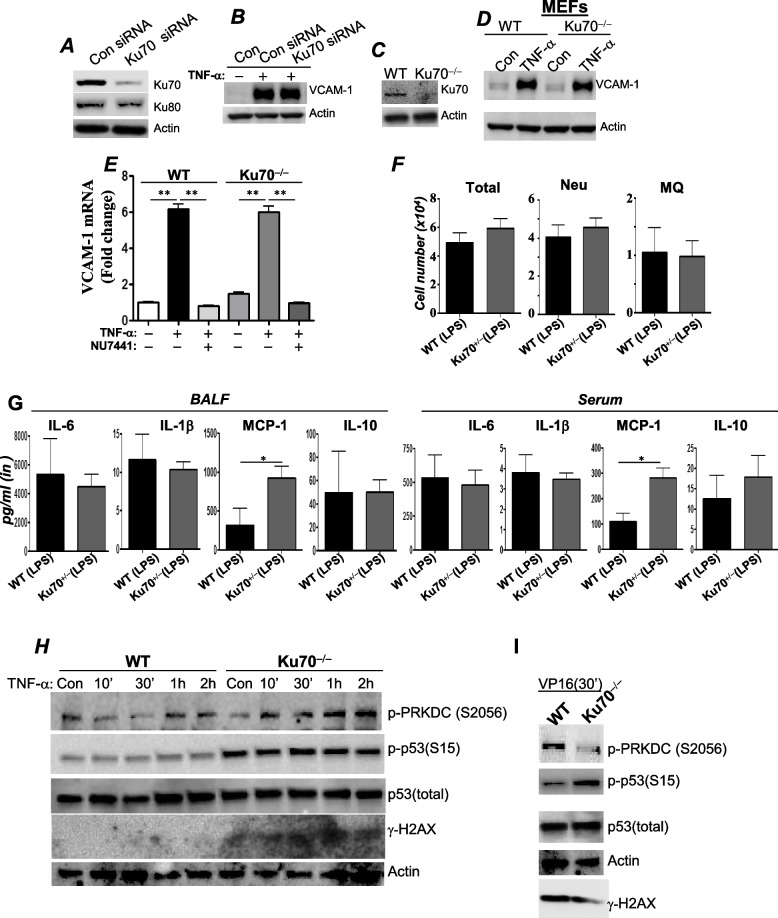
The role of Ku70 in acute lung injury may be dispensable or marginally protective. HAECs were treated with siRNA targeting Ku70 or scrambled siRNA (Con). Protein extracts were subjected to immunoblot analysis with antibodies against Ku70, Ku80 or actin (**A**). **B** These cells were then treated with TNF-α for 18 h. VCAM-1 expression was detected by immunoblot analysis. WT or Ku70^−/−^ MEFs (**C**) were treated with TNF-α for 18 h, after which VCAM-1 expression was assessed by immunoblot analysis (**D**). **E** WT or Ku70^−/−^ MEFs were treated as described in (**D**) but for 6 h in the presence or absence of NU7441. Total RNA was reverse transcribed followed by real-time PCR with primers to mouse *vcam-1*. **, *p* ≤ 0.01; differences from TNF-α-treated cells. **F** WT or Ku70^+/−^ mice were treated with LPS as described in Fig. 1E, and total cell, neutrophil, and macrophage infiltration into the lung was assessed. **G** BALF and sera were assessed for IL-6 and MCP-1 as described above. Data are the means ± SDs of values from *n* ≥ 6 per group. *, difference from LPS-treated WT mice with *p* ≤ 0.05. WT or Ku70^−/−^ MEFs were treated with TNF-α (**H**) or VP-16 (**I**). Protein extracts were assessed for phospho(S2056)PRKDC, phospho-p53(S15), p53, γH2AX or actin by immunoblot analysis with specific antibodies

Ku proteins (Ku70 and Ku80) are prerequisites for PRKDC recruitment to DSBs; thus, cells deficient in either of these proteins are very sensitive to DNA-damaging agents [13, 29]. Mice deficient in Ku70 develop SCID, whereas Ku70^+/−^ mice are normal [9, 31]. Unlike PRKDC deficiency, Ku70 knockdown in HAECs (Fig. 4A) exerted no detectable effect on TNF-α-induced VCAM-1 expression (Fig. 4B). Of note, Ku70 knockdown resulted in a noticeable decrease in Ku80, which is consistent with a previous report [40]. Similarly, Ku70 gene knockout (Fig. 4C) did not influence VCAM-1 expression in TNF-α-treated MEFs at the protein (Fig. 4D) or mRNA level (Fig. 4E). Treatment of Ku70^−/−^ MEFs with the DNA-PK inhibitor NU744 completely blocked TNF-α-induced expression of *vcam-1* (Fig. 4E). These results clearly suggest that Ku70 is dispensable in the regulation of TNF-α-mediated expression of inflammatory factors and that PRKDC is potentially the primary driver of such regulation.

To confirm the independence of PRKDC from Ku70 during inflammation, we examined the effects of partial Ku70 depletion by gene heterozygosity in an LPS-mediated ALI mouse model. It is noteworthy that Ku70 heterozygosity, unlike knockout, does not cause an SCID phenotype in mice, reducing the capacity of cells to respond to irradiation or repair DNA damage [9]. Furthermore, Ku70 heterozygosity does not affect the overall structure of the lung [21]. Figure 4F shows that Ku70^+/−^ mice responded to LPS treatment exactly like wild-type mice. Indeed, the total number of inflammatory cells, including subsets such as neutrophils and macrophages, and their recruitment were unaffected in LPS-exposed Ku70^+/−^ mice compared to their wild-type counterparts. Furthermore, Ku70 heterozygosity did not affect the production of most cytokines (IL-1β, IL-6, and IL-10) in the lungs and sera of animals (Fig. 4G). Interestingly, however, the levels of MCP-1 actually increased in BALF and sera of LPS-treated Ku70^+/−^ mice compared to the wild-type counterparts.

We next hypothesized that if Ku70 is not critical for mounting an inflammatory response, its depletion should not influence TNF-α-induced activation of PRKDC. To test this hypothesis, Ku70^−/−^ MEFs were treated with TNF-α and PRKDC phosphorylation was examined. Figure 4H shows that not only did Ku70 gene deletion not block phosphorylation of PRKDC at S2056, but it actually promoted a slight increase in the modification. As expected, Ku70 gene deletion was associated with a TNF-α-independent increase in p53 phosphorylation and γH2AX levels. Contrary to the treatment of Ku70^−/−^ MEFs with TNF-α, treatment with VP-16 resulted in a decrease in PRKDC phosphorylation (Fig. 4I), which was expected, as Ku70 is a requisite for the DNA-PK response to DNA damage [22].

### p38MAPK regulates TNF-α-induced phosphorylation of PRKDC independently of DSBs through a direct physical interaction and modification

**Fig. 5:**
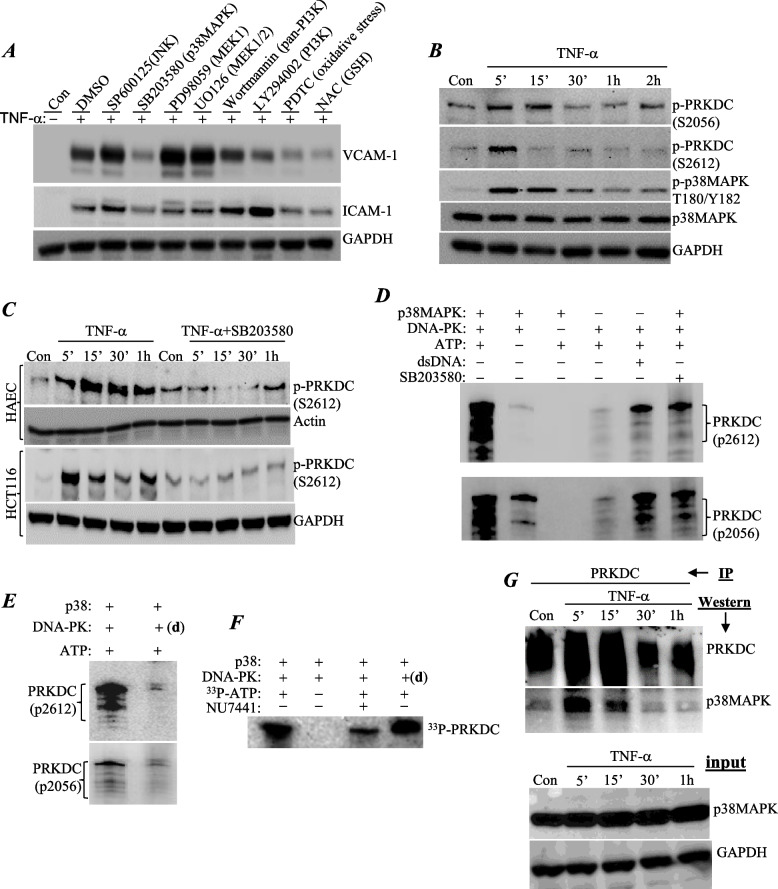
p38MAPK regulates TNF-α-induced phosphorylation of PRKDC independently of DSBs through direct physical interaction and modification. **A** Human endothelial cells were treated with different inhibitors prior to TNF-α exposure and collected 18 h later. VCAM-1 expression was assessed by immunoblot analysis. The inhibitors used were SP60015 (10µM) for JNK, SB203580 (10µM) for p38MAPK, PD98059 (20µM) for MEK1, UO126 (10µM) for MEK1/2, wortmannin (1µM) for general PI3K, LY294002 (1µM) for PI3K, Pyrrolidine dithiocarbamate (PDTC) (10µM) for kinases affected by metal chelation and oxidative stress, and N-acetylcysteine (NAC) (1 mM) for glutathione (GSH)-sensitive kinases. **B** Cells were treated with TNF-α for the indicated time intervals. Protein extracts were examined by immunoblot analysis for phosphoPRKDC at S2056 or S2612, p38MAPK, phospho(T180/Y182)p38MAPK, or GAPDH. **C** HAECs (top panels) and HCT116 cells (bottom panels) were treated with TNF-α in the presence or absence of SB203580 for the indicated time intervals. Phosphorylation of PRKDC and p38MAPK was assessed by immunoblot analysis. **D** p38MAPK (active) and the DNA-PK complex containing PRKDC/Ku70/ku80 (from HeLa cells) were incubated with different reaction components, including ATP, as indicated. Phosphorylation of PRKDC at S2056 or S2612 was detected by immunoblot analysis. **E** p38MAPK (active) was incubated with the DNA-PK complex that was either active or heat-denatured (d) with ATP. PRKDC phosphorylation at S2056 or S2612 was detected by immunoblot analysis. **F** p38MAPK (active) was incubated with the DNA-PK complex either active or heat-denatured (d) with radioactive (^33^P)-ATP in the absence or presence of 2 μM NU7441. Samples were subjected to SDS‒PAGE, and phosphorylation of PRKDC was visualized by autoradiography. **G** Cells were treated with TNF-α after which protein extracts were subjected to IP with antibodies against (total) PRKDC or p38MAPK. Immunoprecipitates were subjected to immunoblot analysis with the indicated antibodies. Input proteins (p38MAPK and GAPDH) are depicted in the bottom panels

A number of kinases have been implicated in TNF-α-mediated signaling [12]. If DNA damage does not occur in response to TNF-α treatment, then it is highly likely that one or more TNF-α-induced kinases are responsible for activating PRKDC. To identify potential candidates, we examined the effect of a number of kinase inhibitors on TNF-α-induced VCAM-1 expression in our cell culture system. p38MAPK inhibition appeared to be highly effective at reducing TNF-α-induced VCAM-1 expression in treated HUVECs (Fig. 5A); this effect on VCAM-1 was confirmed in HAECs (data not shown). Figure 5B shows that the kinetics of PRKDC phosphorylation at the ABCDE (S2612) and PQR (S2056) sites mimicked those of p38MAPK phosphorylation. More importantly, specific inhibition of p38MAPK with SB203580 markedly reduced PRKDC phosphorylation in endothelial (HAECs) and epithelial (HCT116) cells, suggesting a strong regulatory interaction between the two kinases.

Using purified proteins, we assessed whether p38MAPK induces PRKDC phosphorylation. We took advantage of the fact that when active, PRKDC undergoes an autophosphorylation process, which can be assessed by immunoblot analysis. Figure 5D shows that coincubation of active p38MAPK with the DNA-PK complex promoted the phosphorylation of PRKDC at both the ABCDE and PQR sites. To demonstrate that this was an activity of PRKDC rather than p38MAPK phosphorylating PRKDC, we heat-denatured the DNA-PK complex prior to the kinase reaction. The heat inactivation of DNA-PK resulted in complete loss of phosphorylation at the ABCDE and QPR sites (Fig. 5E). To establish that p38MAPK phosphorylated PRKDC in our in vitro system, we used ^33^P-labeled ATP in the reaction with or without the DNA-PK inhibitor NU7441 or heat inactivation. Figure 5F shows that PRKDC phosphorylation was observed both in the presence of its inhibitor and when it was heat-inactivated, demonstrating that p38MAPK did indeed phosphorylate PRKDC.

The above results suggest a likely physical interaction between p38MAPK and PRKDC. To test this hypothesis, we conducted a coimmunoprecipitation assay followed by immunoblot analysis to examine whether TNF-α treatment promoted a direct interaction between the two proteins in endothelial cells. Figure 5G shows that TNF-α promoted a rather rapid (as early as 5 min) but transient physical interaction between PRKDC and p38MAPK. Overall, these results demonstrate an intimate relationship between PRKDC and p38MAPK during TNF-α–induced inflammation.

## Discussion

**Fig. 6:**
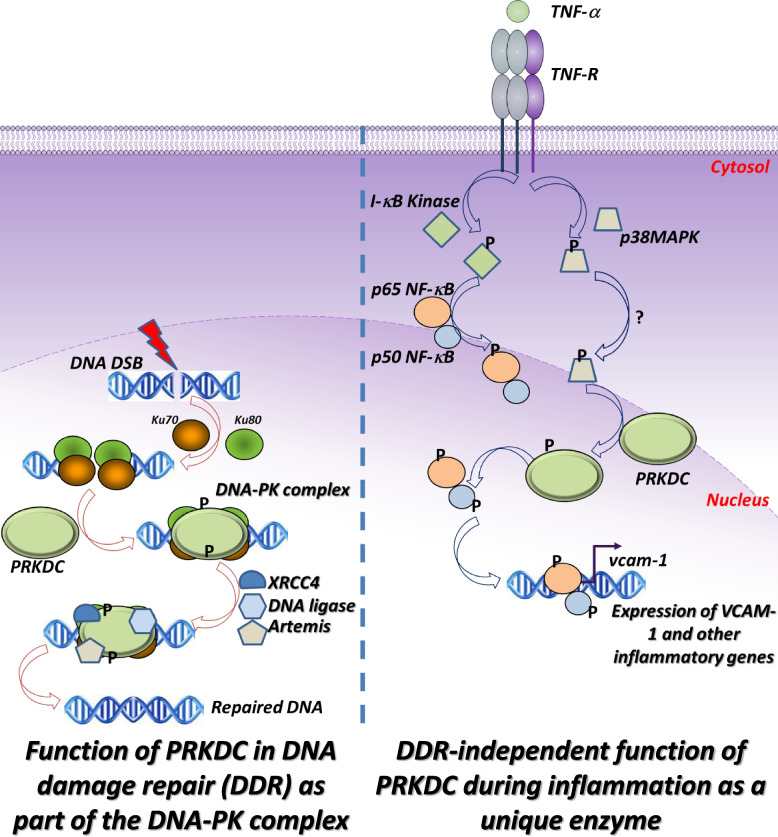
Schematic representation of the novel function of PRKDC as a unique enzyme during inflammation compared to its traditional function in DNA damage repair (DDR). In DDR, Ku70/Ku80 bind directly to DSB leading to the recruitment of PRKDC, which upon a series of phosphorylation, allows for the recruitment of additional DNA repair factors such as Artemis, XRCC4, DNA polymerase, and others to the site of DNA lesion. Such process assure the repair of DSB through the NHEJ pathway. The participation of the Ku proteins is required for the participation of PRKDC in DDR. During inflammation mediated, for instance, by TNF-α, the cytokine binds its receptor (TNF-R), which leads to the activation of a number of kinases such as I- κB kinase (IKK), which lead to the phosphorylation and degradation of I-κBα and the ultimate release of the NF-κB dimer (p65 and p50). The dimer may receive additional modifications (e.g. phosphorylation). TNF-R ligation also leads to the activation of p38MAPK, which, according to our results, rapidly phosphorylates PRKDC independently of the DDR machinery. Activated PRKDC phosphorylates serine 20 of p50 NF-κB, which allows for the final binding of the transcription factor to promoters of target genes (e.g. *vcam-1*) and expression of mRNA. Translation of these mRNAs lead to production of proteins that participate in the manifestation of inflammation. In this scenario, PRKDC functions as a unique and DDR-independent kinase hence our proposal to name it DNA Repair-Independent Inflammation-dependent Protein kinase (DRID-PK)

The present study revealed a completely novel aspect of DNA-PK function independent of its canonical role in DNA repair (see scheme in Fig. 6). We have established a very strong unprecedented relationship between TNF-α, PRKDC and p38MAPK, which may explain the DNA repair-independent function of the kinase during inflammation. We demonstrated that TNF-α is a potent activator of PRKDC, as it promotes its autophosphorylation very rapidly as well as its interaction with p38MAPK. We have demonstrated the direct phosphorylation of PRKDC by p38MAPK independent of DNA damage. We have identified new sites (data not shown) that may be critical for future studies on the role of PRKDC in inflammation, which will allow for the development of new drugs that can block kinase function during inflammation without affecting DNA repair. We have demonstrated a disconnect, perhaps an antagonism, between PRKDC and Ku70, clearly suggesting that the catalytic subunit functions independently of Ku70 and that it may constitute a novel enzyme during inflammation. It is important to note that PRKDC cannot be considered an enzyme during the DNA repair process, as its function is strictly dependent on Ku70 and Ku80 as part of the DNA-PK complex. It is also important to mention that there is a strict interdependence between Ku70 and Ku80, as both subunits have to be present for the DNA-PK complex to be functional. Such uniqueness and its independence of DSBs [22] merit considering a change in its nomenclature to separate its radically different role as a complex trimer (PRKDC/Ku70/Ku80) in DNA repair from its role as an individual enzyme (PRKDC only) in inflammation. We propose naming the new kinase DNA Repair-Independent Inflammation-dependent Protein kinase (DRID-PK). Obviously, this is a mere suggestion of change in nomenclature, which needs to be validated by independent research reports.

One of the biggest challenges in inflammation and the development of strategies to block or mitigate morbid aspects of associated diseases is the identification of specific targets whose modulation does not lead to undesired side effects. This is simply because most of the targets have beneficial physiological roles in cells and tissues. Accordingly, the issue of finding a target that may be “dispensable” is a monumental task and difficult to achieve. Our laboratory has made tremendous effort in the last two decades to understand the roles of DNA repair enzymes, namely, PRKDC and poly(ADP-ribose) polymerase (PARP)-1, in inflammatory and immune responses. Initially, we speculated that the relevant functions of these enzymes were strictly linked to DNA damage potentially caused by oxidative stress. However, as we gained more knowledge, it became very clear that the role of these enzymes might be completely independent of DNA repair [7, 32]. This may be because enzymes are highly abundant in cells and that the DNA repair process requires minute levels of the enzymes. What is exciting about this is that partial inhibition, pharmacologically by low to moderate doses of drugs or by gene heterozygosity, which reduces the expression of proteins by ~ 50%, provides outstanding protection against inflammation. Recently, we showed that PARP inhibitor-based metronomic therapy (sub-IC50 concentrations or doses at repeated intervals) or gene heterozygosity, which does not interfere with DNA repair or induce the STING pathway, blocks the suppressive function of myeloid-derived suppressor cells (MDSCs), allowing CD8^+^ T cells to attack tumors and synergize with anti-PD-1 immunotherapy in colon cancer models [7]. We also showed many years ago that partial inhibition of PARP-1 reduces atherosclerotic burden and promotes plaque stability and regression of established plaques in animal models of the conditions [10, 11, 32] (also see editorial [18]). Based on the findings of this study and our previous reports, we propose the new concept of “dispensability of therapeutic targets”. This basically means that inhibiting or reducing most of the target either by enzymatic inhibition or by targeting its integrity by promoting its degradation through proteolysis targeting chimeric (PROTAC) technology [37] or synthetic degradation [41] would not affect the normal function of the enzyme, and thus, side effects may not ensue. It is also important to note that in these situations, complete inhibition or elimination of the target is not necessary, as the role of these enzymes in inflammation is highly sensitive to an inhibition of its enzymatic activity or its protein levels. This is obviously very different from what is known as synthetic lethality mediated by the complete inhibition of PARP in BRCA-deficient cancers [26]. To achieve synthetic lethality, high doses of PARP inhibitors are necessary.

In the present study, we demonstrate that p38MAPK regulates PRKDC by physically interacting with and phosphorylating the kinase as well as by inducing autophosphorylation at both the ABCDE and QPR sites. While phosphorylation of PRKDC on the ABCDE cluster (e.g., at S2612 or T2609) promotes access to DNA breaks, phosphorylation of the PQR cluster (e.g., at S2056) is predicted to limit access of the kinase to DNA ends [23]. It is tempting to speculate that phosphorylation at the PQR cluster (e.g., at S2056) of PRKDC allows for recruitment of the kinase in inflammatory responses, as it is predicted to inhibit access of the kinase to DNA ends [23]. However, our results show that these two sites are often phosphorylated simultaneously, which refutes this possibility. Accordingly, it is rather premature to speculate on the regulatory mechanisms without additional studies and new tools. The possibility of other kinases regulating PRKDC is highly likely. Many stimuli induce the p38MAPK pathway [3]. It would be interesting to unravel more unique pathways that regulate PRKDC function. We hope the present study will be a platform on which other investigators discover new DNA repair-independent regulators of PRKDC. A physical interaction between p38MAPK and PRKDC implies that the two proteins should be in the same subcellular compartment at some point after TNF-α stimulation. This may constitute a conundrum. An important question becomes evident: how could a cytosolic kinase (p38MAPK) interact with a nuclear kinase (PRKDC)? Although p38MAPK is primarily cytoplasmic, it was shown to translocate to the nucleus [39, 44]. The cytosolic localization of PRKDC was reported [5, 14] but has been highly contested. Huston et al. [14] showed that EPAC coupled to the GTPase Rap2 can regulate the subcellular localization of PRKDC and promote its cytosolic localization, which is counteracted by PKA [14]. Interestingly, immunofluorescence with antibodies against the QPR phosphorylation site revealed the cytosolic localization of activated PRKDC in human atherosclerotic plaques (Ghonim et al*. manuscript in preparation*). More detailed experimentation is needed to decipher the connection between p38MAPK and PRKDC. It is important to acknowledge that p38MAPK is not strictly related to TNF-α-mediated signaling as it can be regulated by numerous stimuli [3] and the dysregulation of its associated pathways have been strongly linked to various inflammatory disorders [3]. This includes acute lung injury [20], chronic airway inflammation [28], as well as pulmonary fibrosis [34], suggesting the possibility of targeting the enzyme as a viable therapeutic target for mitigating the progression of inflammation.

The pathology associated with Ku70 (or Ku80) deficiency may be linked to an accumulation of DNA damage in cells and tissues. The persistence of such deficiency is predicted to increase promotion of inflammatory factors. Interestingly, our results show that Ku70 heterozygosity failed to block allergen (ovalbumin)-induced hyper-responsiveness (AHR) in an animal model of asthma (Supplemental Fig. S4). This suggests that a more severe depletion of the Ku70 is needed to cause that intra and extracellular damage necessary to cause disease observed by Rehmen et al. [33] and Nog et al. [30]. It is, therefore, tempting to speculate that Ku70 may exert an antagonistic function against PRKDC during inflammation and lends more support to the notion that the two proteins do not work in concert during inflammation and that PRKDC is a unique inflammatory enzyme. The disconnect between DNA repair and inflammatory processes was strengthened by our results showing that when the two processes are combined, DNA damage tends to block the expression of inflammatory factors such as VCAM-1 (Fig. 3H).

## Conclusions

The results of the present study allow the establishment of a completely novel and unprecedented DNA repair-independent function for PRKDC in the pathogenesis of inflammation and determine the mechanism(s) by which the kinase plays a critical role in inflammation. This study puts forth a very novel and paradigm-shifting concept that challenges the current understanding of the role of PRKDC in cellular and tissue processes and may allow us to consider the subunit as a new and distinct enzyme. Unraveling the mechanism by which the kinase functions during inflammation and identifying the differences in its function in DNA repair provides a platform on which new drugs can be developed in such a way that DNA repair processes are not interfered with while blocking inflammation. More importantly, given the high sensitivity of PRKDC to inhibition during inflammation, targeting PRKDC would not affect DNA repair. It is important to state that targeting DNA repair in nonneoplastic diseases is faced with a great deal of skepticism because of the perception of causing DNA damage deficiency-associated side effects; thus, targeting its dispensability may dampen such skepticism and open new opportunities for the discovery of novel therapies.

### Supplementary Information

**Supplementary Material 1. **

## Data Availability

N/A.

## Abbreviations

ALI: Acute lung injury
BALF: Bronchoalveolar lavage fluid
DHT: Delayed hypersensitivity
DNA-PK: DNA-dependent protein kinase
DSBs: Double stranded DNA breaks
DRID-PK: DNA repair-independent inflammation-dependent protein kinase
MEFs: Mouse embryonic fibroblasts
MPO: Myeloperoxidase
PARP-1: Poly(ADP-ribose) polymerase-1
PRKDC: Protein kinase, DNA-activated, catalytic subunit
SCID: Severe combined immunodeficiency
V(I)CAM-1: Vascular (intercellular) adhesion molecule-1
