# Supplementary material for: Unconventional activation of PRKDC by TNF-α: deciphering its crucial role in Th1-mediated inflammation beyond DNA repair as part of the DNA-PK complex
Source: J Inflamm (Lond). 2024 Apr 30;21:14. doi: 10.1186/s12950-024-00386-x (PMC11059672; doi:10.1186/s12950-024-00386-x)
Supplement: Supplementary file 1 — Supplementary Material 1. [file 12950_2024_386_MOESM1_ESM.pdf]

Supplementary data

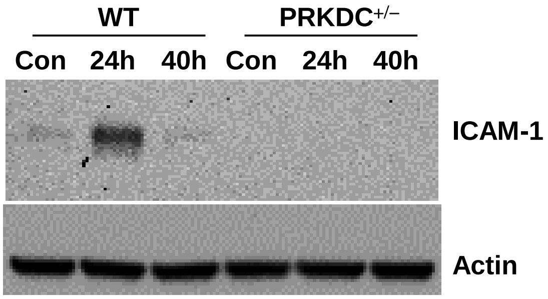

Fig. S1. ***The absence of ICAM-1 expression in TNF-α-induced PRKDC$^{+/-}$ cells is not simply delayed as the effect is still visible even after 40 h of treatment with the cytokine***. Cells were treated with TNF-α for 24 or 40 h. Cell extracts were assessed for ICAM-1 by immunoblot analysis with antibodies to ICAM-1 or Actin.

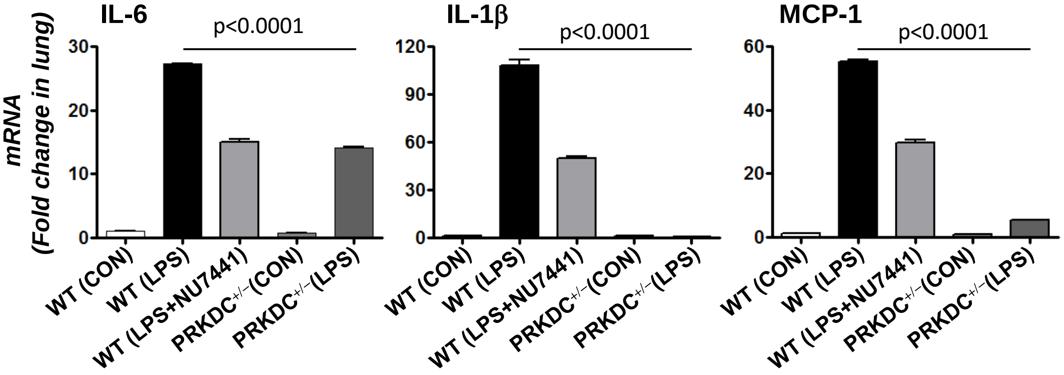

Fig. S2. ***PRKDC heterozygosity or treatment with NU7441 reduced expression of IL-6, IL-1β, and MCP-1 but not IL-10.*** The absence of ICAM-1 expression in TNF-α-induced PRKDC$^{+/-}$ cells is not simply delayed as the effect is still visible even after 40 h of treatment with the cytokine. Cells were treated with TNF-α for 24 or 40 h. Cell extracts were assessed for ICAM-1 by immunoblot analysis with antibodies to ICAM-1 or Actin.

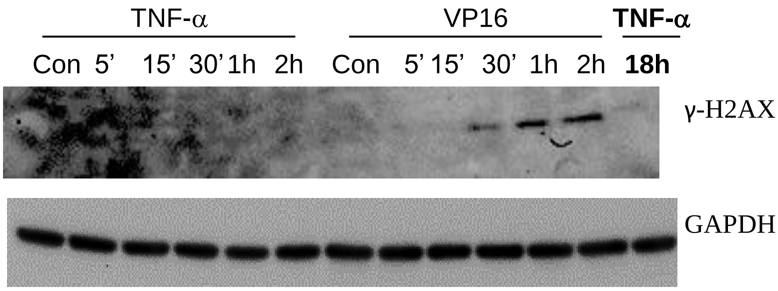

Fig. S3. ***Treatment with TNF-α does not induce DNA damage response even after 18 h of treatment in endothelial cells.*** Cells were treated with TNF-α or VP-16 for the indicated times. Cell extracts were prepared and subjected to immunoblot analysis with antibodies to γH2AX or GAPDH .

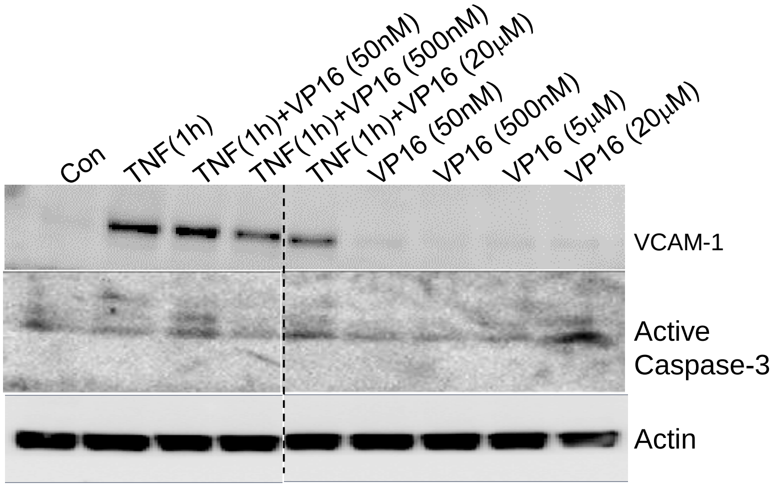

Fig. S4. ***Treatment with TNF-α does not induce DNA damage response even after 18 h of treatment in endothelial cells***. Cells were treated with TNF-α or VP-16 for the indicated times. Cell extracts were prepared and subjected to immunoblot analysis with antibodies to γH2AX or GAPDH .

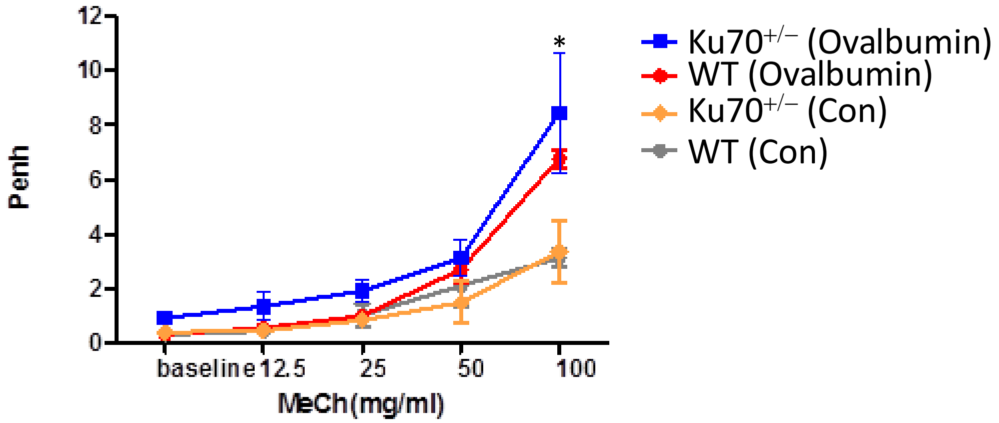

Fig. S5. ***Ku70 heterozygosity does not protect against airway hyperresponsiveness in response to ovalbumin in a mouse model of asthma***. Mice were sensitized and challenged followed by assessment of airway hyperresponsiveness through a measurement of enhanced pause (Penh) using whole body plethysmography essentially as described (Ghonim, Pyakurel et al. 2015). Penh was recorded 24 h after a single ovalbumin challenge upon exposure to the indicated concentrations of aerosolized methacholine (MeCh). Results are plotted as maximal fold increase of Penh relative to baseline and expressed as mean ± SEM where n=6 mice per group. *, difference from control mice, $p < 0.05$.
